# Solvent Properties of Buffalo Lithia Water

**Published:** 1896-12

**Authors:** 


### Selections and Abstracts.

---

### SOLVENT PROPERTIES OF BUFFALO LITHIA WATER.

George Halstead Boyland, M. A., M. D., member of the Faculty of Paris, France, (formerly Professor in Baltimore Medical College, etc.,) begins a paper on this subject (*New York Medical Journal*, August 22, 1896) with the statement that in no department of therapeutics have so great services been rendered as in the treatment of disease by mineral waters. It is a pleasure to note that, notwithstanding the author's residence abroad for years, he has not forgotten the value of American products, as so many who go into foreign countries do; for he states that experience has taught that we must prescribe the Buffalo Lithia waters of Virginia if we wish to dissolve concretions from the blood and different viscera, and prevent their reformation in the sanguineous fluid, as also their re-deposit in the organs of the body. This remedy is so subtle in its composition and yet so sure in its working as to excite the wonder of the most profound chemist and the most skilful practitioner. If the component parts of these waters be used in precisely the same proportions as to weight and chemical purity, in compounding in water, presenting an exact reproduction of the natural water, by some mysterious process of Nature their solvent power is diminished or reduced to zero.

Still more inexplicable is the fact that the salts of the Buffalo Lithia waters act with rapidity in dissolving uric acid and other calculi—whether hepatic, renal or vesical—in very weak solutions, while stronger ones are less active or fail altogether. Dr. Roberts (London) gave valuable data on this subject (*Arch. of Medicine*, Vol. iii.). He placed fragments of uric acid calculi (weighing 40 to 112 grains each) in ten-ounce vials of carbonate solutions of various strengths, analogous to that of Buffalo Lithia water, Spring No. 2. The experiments continued day and night for some time, the daily flow of water varying between six and fifteen pints. Above a strength of 120 grains to the pint no solvent power was exerted; even with 80 grains to the pint there was only a little. In solutions of 60 grains to the pint, the solvent action was more noticeable; still more so in solutions of 50 grains to the pint; at 40 grains to the pint the activity was further increased; and, so on down, the solvent action became stronger and more speedy as the strength of the solution approached the quantities contained in the waters of Spring No. 2, which is justly considered to be the most powerful and perfect solvent of the various forms of calculi known to science; and not of calculi only—whether red lithic or uric acid, or the triple phosphate of ammonium—magnesium variety—but also of morbid concretions in general, such as albumin, sugar, cholesterin crystals, etc.

Years ago, when Dr. Boyland was resident physician at these world-renowned Springs, a patient with red uric acid kidney calculi, to assure himself of the solvent potency of Buffalo Lithia waters, placed a piece of calculus (about the size of the head of a large pin), passed by himself, in a goblet of water from Spring No. 2, leaving the same covered on a shelf in his bedroom. Two weeks later the calculus had entirely disappeared in solution. To verify the accuracy of this important statement, Dr. Boyland placed ten grains of a red lithic-acid calculus, and an equal quantity of a triple phosphatic deposit of whitish ammonium-magnesium formation, side by side, in separate glass receptacles, each containing thirty ounces of Buffalo Lithia water, Spring No. 2. These vessels, covered with glass, were left standing, high out of reach.

At the end of two weeks, disintegration of the *red uric or lithic-acid formation* had begun; a week later this calculus had separated into three fragments, which in another eight days presented a clean water-worn appearance, with a few loose flakes floating on the surface of the water. At the end of six weeks the solution was complete—all traces of the stone having disappeared.

With the *whitish-gray triple-phosphate of ammonium-magnesium* deposit, the process was somewhat different, but solution was accomplished in the same length of time. Here a whitish coating formed around the original calculus which softened it, causing it to break into many pieces resembling very coarse sand, and finally to dissipate itself, and vanish altogether.

The same experiment was then made with the water of Spring No. 2 so arranged, by means of a spigot and retorts, as to be continually flowing, thus constantly washing the same amount of the same varieties of calculi in the bottom of the retorts. Exactly the same solvent process took place, except that all traces of either stone disappeared in thirty days instead of six weeks.

Analogous experiments with the waters of Spring No. 1 showed that at the end of thirty days dissolution of both varieties of calculi was complete, although the water acted a little more promptly in dissolving calculi of triple phosphate of ammonium-magnesium than those of uric-acid concretion.

The water of Spring No. 3 has also a solvent potency, but in a comparatively feeble degree.

But the conclusion from the experiments with the waters derived from Springs Nos. 1 and 2 was that they possess a *solvent power superior to any known mineral water.*

Professor Tonry's analysis (expressed in grains to the Imperial gallon) shows that Spring No. 2 contains—

29,300 grains of carbonate of potash.
2,250 grains of bicarbonate of lithium.
4,921 grains of chloride of sodium.

—a mixture of such nice proportions, when combined with the other ingredients, as to surpass the most ingenious formulæ of the materia medica.

Coming to the solvent action of Buffalo Lithia water inside the human body:—Dr. J. Milner Fothergill, recognized as authority the world over, said (*Handbook of Treatment*) with reference to liver and kidney affections, especially where uric acid and lithæmia are prominent symptoms, the sovereign remedies are: "Soda and lithia for the liver; lithia and potash for the kidneys."

Dr. Boyland's experience and observation, covering many thousand cases at the Springs, in different parts of the United States and in Europe, confirm what he long ago said, that in cases in which lithia, soda and potash are specially indicated, he has obtained far better results from the Buffalo Lithia waters than from any of the preparations of lithium; furthermore, there is no mineral water in America or Europe so singularly adapted to such a large number and variety of maladies.

The solvent properties of all three Springs on grape sugar is immediate (as shown by placing ten or twenty grains in a test tube containing half an ounce of water); and their value in *diabetes mellitus* is attested by numerous cases. Although the solvent properties of these waters may be less evident *in vitro* as regards albumin, that exercised upon albumin *in corpore* and in blood in albuminuria and icteric fever, which it dissolves as it does biliary elements and cholesterin crystals contained therein in the latter disease, is as remarkable as it is in lithæmia, lithiasis, and different forms of hepatic, renal and vesical calculi. Albumin, biliary constituents, such as cholesterin crystals and gallstones, sugar, uric acid, and phosphatic calculi, are characteristic of so great a variety of diseases that they may be said to dominate all pathology; and it is because of the active solvent potency upon these different morbid concretions that the Buffalo Lithia waters are unrivalled in the treatment in so wide a range of pathological processes.

As illustrative cases, notes are made of the following:

I. *Nephritic Colic.*—Mr. A. had frequent attacks of nephritic colic; came to Springs with constant lumbar pains, passing quantities of red uric gravel, varying in size from pin-head to a large pea; florid sanguineous temperament; Heberden's nodosities on fingers; tongue heavily furred, etc. Six weeks' course of Spring No. 2 water cured him.

II. *Triple Phosphate of Ammonium-Magnesium Calculi.*—Mr. G., small stature, emaciated, pale, almost continuously passing calculi, with vesical pain and tenesmus of sphincter; urine mixed with blood and mucus. Began upon three glasses a day of Spring No. 1, increased gradually to six, then to eight. Decided improvement in ten days—pain much alleviated; no blood or mucus in urine. A week later, calculi became like sand; ten days later all disease symptoms had vanished. He was now put on three glasses a day of Spring No. 3 to prevent reformation of calculi and to overcome anæmia.

III. *Gallstones.*—Mrs. L. had exhausted usual remedies with out relief. Attacks had been succeeding each other every few days. Owing to gastritis, only half glass of Spring No. 2 water, four times daily, could be used at first. In two days gastric symptoms were relieved, and the dose of water was rapidly increased. She had only one attack after arrival. In eight weeks she left the Springs—jaundice having given place to healthy clearness and rosy cheeks.

IV. *Suppressed Gout.*—Excess of uric acid in urine; slight tophous roughness along borders of helix; some emaciacion. Four glasses a day of Spring No. 2, for four weeks, rendered urine normal; he had gained four pounds in weight.

V. *Gout.*—Large, plethoric woman, weight about 200 pounds; left Springs after five weeks' course of Spring No. 2, much benefitted and 20 pounds less weight. Urine free from uric acid; no crystals after second week; chalky deposits and tophi in joints considerably reduced in size—smaller ones completely resorbed.

By their solvent power, these waters accelerate excretions, promote nutrition and assimilation, and thereby regulate the proportion of adipose to muscular tissue.

*Lithæmia* is the dyscrasia of calculus or gravel; *lithiasis* the complication of calculi themselves. The term may be practically applied to all cases of spurious or abortive gout, hepatic, renal and vesical disease characterized by mixed lithic formations, not described in pathological nomenclature, nor belonging to any regular pathological or anatomical class. The waters of Spring Nos. 1 and 2 act with equal promptness in in such cases, purifying the blood by dissolving the lithic elements contained therein, preventing their reformation in lithæmia and dissolving the calculi in lithiasis.

*Stone in the bladder*, whether of renal or vesical origin, and whether of oxalic, uric or phosphatic composition, is an iudication *par excellence* for the use of Buffalo Lithia Waters. Perhaps the most striking of cured cases was the following:—

VI. *Triple Phosphate Vesical Calculus.*—Mr. C., of N. C., came to the Springs with a large single vesical calculus. Under the use of the water the stone disintegrated into smaller fragments; these, in turn, into gravel, and that eventually into sand, until, finally, there was no discharge of calculus concretion in any form; the urine, from having been strongly ammoniacal, became perfectly normal. There was no return of the disease a year later.

*Jaundice*—a common complication in hepatic disease—is quickly relieved by these waters, which dissolve cholesteric crystals and alkalize the blood.

*Albuminuria.*—No remedy is so absolutely specific in all forms of albuminuria and Bright's disease—acute or chronic—as Buffalo Lithia water, Spring No. 2, accompanied by a milk diet. In the *albuminuria of pregnancy*, if this water is used even as late as the last week before confinement, albuminuria disappears, and the woman is almost guaranteed against *puerperal convulsions.*

*Diabetes and glycosuria.*—Dr. Boyland has treated a number of cases with these waters, where patients persevered in treatment, with perfect cures in some; marked benefit in others. He relies upon Springs Nos. 1 and 2 at times singly; at others, in combination for the main treatment; and on Spring No. 3 as an adjuvant and chalybeate to overcome the usual anæmia. The solvent action is here most marked, not only as regards the glycogen in the blood, but also hippuric acid contained therein, and which Prout and Garrod first found in diabetin urine (Beale, *Urinary Deposits*).

VII. *Fat Diabetes* (*Diabetes Gras*).—Mr. B., Baltimore, on arrival at Springs, his urine (sp. gr. 1.045) contained three drachms of sugar to the quart. He had marked polyphagia, polydipsia, polyuria. During the first four weeks he was put upon the water of Spring No. 2; during second four weeks upon that of Spring No. 1. Quantity of sugar diminished from beginning; appetite and thirst lessened, until end of eighth week, when all diabetic symptoms disappeared. Treatment was continued two weeks longer—two glasses of Spring No. 1 and two of Spring No. 3 were drunk each day. He was completely restored to health.

VIII. *Thin or Pancreatic Diabetes.*—Miss——, age 17. Nervous crises, lumbar pains, wasting, urine loaded with sugar; symptoms of pancreatic diabetes. Two weeks after she began taking water of Spring No. 2 sugar in urine greatly diminished; nervous crises ceased, as also lumbar pains. She now left the Springs and the case was lost sight of.

*Accidental Glycosuria, without Diabetic symptoms*, is usually cured by Buffalo Lithia water in a few days—sometimes in twenty-four hours.

Where *Appendicitis* depends on the formation of phosphatic deposits in the appendix, the waters of Springs Nos. 1 and 2 will prevent the reformation of calculus after operation. Phosphatic appendicitis is a danger which patients with lithic acid diathesis always run. A case of appendicitis at the Springs made a good recovery without operation, drinking of Spring No. 1 throughout the duration of the disease.

To sum up:—Springs Nos. 1 and 2 possess equally solvent power, which makes these waters superior to all others, not excepting the waters of Carlsbad. He prefers Spring No. 2 in the red lithic or uric acid dyscrasia; and Spring No. 1 in triple-phosphate of ammonia magnesia—although perhaps No. 2 is as good. Spring No. 3 is an excellent adjuvant in the anæmia of albuminuria, diabetes, etc.

As Springs Nos. 1 and 2 hold the same mineral constituents, the change in their composite quantities probably makes the difference in indication for their use. These waters are equally active and efficacious remote from the Springs—the only advantage derived from drinking them *in loco* being due to change of air, scenery, cooking, etc. He regards Buffalo Lithia waters as the most speedy, radical and permanent cure of the greatest variety of cases in which lithia, potash and soda are especially indicated of any mineral waters prescribed by themselves. He considers the salts of lithium, as held in natural solution in these waters, far more efficacious than any pharmaceutical preparation of those salts.—*Virginia Medical Semi-Monthly.*

